# Determinants of fatigue and stress

**DOI:** 10.1186/1756-0500-4-238

**Published:** 2011-07-20

**Authors:** Rüya D Kocalevent, Andreas Hinz, Elmar Brähler, Burghard F Klapp

**Affiliations:** 1 Department of Mental Health, University of Leipzig, Germany; 2 Department of Psychosomatis, Charité University Medicine Berlin, Germany; 3 Department of Medical Psychology and Medical Sociology, University of Leipzig, Germany

**Keywords:** Fatigue, stress perception, epidemiology, health determinants

## Abstract

**Background:**

Fatigue can be triggered by previous perceived stress which may lead to impairment of performance and function. The purpose of the study was to investigate the relationship between fatigue and perceived stress.

**Method:**

Health determinants including sociodemographic factors for associations between fatigue and perceived stress in the general population (N = 2,483) are outlined. Fatigue and stress were assessed with the Chalder Fatigue Scale (CFS) and the Perceived Stress Questionnaire (PSQ).

**Results:**

Within the general population, 25.9% of male and 34.5% of female respondents reported moderate fatigue during the last six months; 9.7% of subjects reported substantial fatigue lasting six months or longer. An adjusted regression analysis (R^2^corr = .28, p < .001) showed that fatigue is highest associated with perceived stress and self-perceived health status. The following factors were correlated with increased rates of fatigue and perceived stress: female gender, divorce/separation, low social class and poor health status.

**Conclusion:**

We conclude that the two conditions overlap most in terms of socio-economic status and self-perceived health status.

## Background

Unexplained fatigue is a relatively common condition in the community and primary care [1].

A recent study in the Netherlands has reported a prevalence rate of fatigue up to 36.4% in the general population associated with unhealthy lifestyles or coping mechanisms [2]. That corresponds with results from the *Eurobarometer Mental Health*, where 26% of the respondents from 27 European countries reported to be "tired most of the time" and only 51% felt "full of energy" [3]. There are several studies showing that fatigue is associated with psychiatric morbidity, especially depression [1,4-7]. Yet fatigue can also be triggered by previous perceived stress which may lead to impairment of performance and function [8,9]. Higher self-reported stress in the premorbid period is associated with higher risk for chronic fatigue (odds ratios, 1.64-5.81) [10]. Stress, including psychological stress, can also have a biopsychosocial influence on fatigue [11]. A study of patients with chronic fatigue syndrome showed that the hypothalamo-pituitary-adrenal (HPA) axis component of their stress response was dysfunctional [12]. But the nature of this association is still not clear.

In order to detect the relation of fatigue and perceived stress it can be argued what distinguishes perceived stress from anxiety and depression. In former studies recent perceived stress (last 4 weeks) could be distinguished from state but not from trait anxiety. In terms of depression results vary between *r = *0.49-0.56 [13-15].

To our knowledge, there have been only a few nationally representative studies of stress perception in the general population [15,16]. This lack is the result of several factors, including the challenge of operationalizing the relation or process of fatigue and stress perception. This paper reports findings from the Chalder-Fatigue-Scale (CFS) and the PerceivedStress-Questionnaire (PSQ) in the general population. We aimed to:

(1) report the prevalence of fatigue and perceived stress, as well as associated sociodemographic risk factors in a nationally representative sample, and (2) determine the overlapping correlates of fatigue and perceived stress.

## Methods

A nationwide survey representative of the German general population was conducted with the assistance of an institute certified for demographic research (USUMA, Berlin) according to the German law of data protection (§30a BDSG) and with written consent. Previously ethics were weighted to the respective interests of the public and of the individuals concerned following §823 (BGB) of the Civil Code of Law and in accordance with the guidelines in the Declaration of Helsinki. Age, gender, and educational level were the major criteria for representativeness according to the register of the 1994 Federal Elections. The response rate was nearly 80%. The set of questionnaires was administered to the final sample of 2483 persons. In the first wave, attempts were made to contact 3125 persons. Two callbacks had to be without success before an address was considered a failure. The three-stage sampling procedure consisted of sample points in the first, household in the second, and persons in the third stage. Target households within the sample points were determined using the random-route procedure; the target persons within the households were selected using random digits. The basic population for the data collection is made up of the German population aged at least 16 years and living in private households. The data sets for individuals aged at least 18 years were used for the data evaluation.

### Assessment of Fatigue (CFS)

The CFS was developed to measure the severity of fatigue [17] and has been used in several studies. The 11-item scale was found to be reliable (Physical Fatigue: *r *= 0.85; Mental Fatigue: *r *= 0.82; Total Score: *r *= 0.89) and valid. Fatigue is defined as a continuous dimension as opposed to a category. Response options include: 0 = 'better than usual', 1 = 'no more than usual', 2 = 'worse than usual' and 3 = 'much worse than usual'. Symptoms that are not related specifically to fatigue but that are associated with the chronic fatigue syndrome were not included since the intention was to produce a scale that measured fatigue specifically.

### Assessment of Perceived Stress (PSQ)

The construct validity of the PSQ was tested within a structural regression model [18]. The German and international applications of the PSQ and descriptive indicators of the sample are described in greater detail elsewhere [16,18]. The PSQ includes 30 items that are assigned to seven scales (Harassment, Overload, Irritability, Lack of Joy, Fatigue, Worries and Tension). The items refer to the period of the last four weeks (recent version) and can be answered with a 4-point rating scale (1 = almost never, 2 = sometimes, 3 = often and 4 = usually). The resulting PSQ Total Score was linearly transformed between 0 and 1 according to Levenstein et al. (PSQ = (raw value - 30)/90) [13]. The Cronbach's α value of the PSQ is high (α = 0.93).

### Analytic strategy

The prevalence rates that are presented here were calculated by an analysis of frequencies. Logistic regression (odds ratios and 95% confidence intervals) was used to quantify the associations between fatigue, stress perception and their sociodemographic correlates. Linear regression was used to quantify the association between fatigue and perceived stress. The data were analyzed with SPSS (Version 18.0).

## Results

### Description of the sample

The 2,483 subjects who took part in the study were similar in sex distribution (52.7% female/n = 1346, 47.2% male/n = 1206) to the original sample of N = 3125 with a mean age of 47.6 years (SD 18.0). 52.2% were married, 24.4% single, 1.1% married but separated from the partner, and 22.1% divorced or widowed, and 14.5% were unemployed.

### Overall prevalence

Among the general population, 9.7% of individuals reported substantial fatigue lasting six months or longer; 25.9% of male and 34.5% of female respondents reported fatigue during the last 6 months.

The PSQ Mean Score was 0.30 (SD 0.15). Thus the cut-off score for moderate level of perceived stress was estimated to be > 0.45 to ≤0.60 and for high level 0.60. By using the cut-off scores described below, the prevalence of perceived stress at a moderate level was estimated to be 14.5%. The prevalence of perceived stress at high levels was 3.1%.

### Relation between Perceived Stress and Fatigue

Table 1 shows the correlation between fatigue and the seven scales of the PSQ. The total score of the CFS and the Fatigue subscale of the PSQ showed the highest association (*r *= 0.48), followed by the Lack Of Joy subscale (*r *= 0.39) and Tension subscale (*r *= 0.35). Except for the correlation between the total score of Physical Fatigue and Overload (*r *= 0.02), all of the scales were associated on a p < 0.05 level. A regression analysis (R^2^corr = .28, p < .001) showed that fatigue is highest associated with perceived stress (β = 0.30) and self-perceived health status (β = -0.27), adjusted for age, gender, marital-, socioeconomic-, and employment status.

**Table 1:** Correlation matrix for the CFS and the PSQ

2 Scales of Fatigue					7 Scales of the PSQ						
	**Fatigue** **Total**	**Mental** **Fatigue**	**Physical** **Fatigue**	**PSQ-Total**	**Harassment**	**Overload**	**Irritability**	**Lack of Joy**	**Worries**	**Tension**	**Fatigue**

**Fatigue** **Total**	1.00	0.88**	0.97**	0.37**	0.15**	0.05*	0.26**	0.39**	0.32**	0.35**	0.48**
Mental Fatigue	-	1.00	0.73**	0.33**	0.16**	0.07**	0.23**	0.34**	0.29**	0.31**	0.38**
Physical Fatigue	-	-	1.00	0.35**	0.12**	0.02	0.24**	0.38**	0.29**	0.33**	0.48**

Correlation coefficient: *Spearman's r*.

*p < 0.05

**p < 0.001.

### Sociodemographic and health related odds ratios of perceived stress and fatigue

The significant sex differences were indicated in table 2: women have a higher risk of fatigue and higher rates of stress perception. Age had a different effect. The elderly (over 61 years) had the highest risk of fatigue, while the maximum odds ratios for stress occur in those between the ages of 41 years and 60 years.

**Table 2:** Sociodemographic and health related odds ratios of perceived stress and fatigue

	Prevalence of Stress	Odds Ratios^1^	95% CI	Prevalence of Fatigue	Odds Ratios	95% CI
	**n (%)**			**n (%)**		
**Sex**						
Men	8.5%	-	-	25.5%	-	-
Women	11.2%	1.25*	1.03-1.53	33.7%	1.49**	1.24-1.78

**Age**						
16-40	19.7%	1.18	0.92-1.52	19.6%	-	-
41-60	22.2%	1.37*	1.06-1.77	31.3%	1.87**	1.48-2.36
61 >	17.2%	-	-	42.0%	2.97**	2.37-3.73

**Marital Status**						
Married	231 17.7%	-	-	29.3%	0.64**	0.51-0.79
Single	120 19.8%	1.14	0.90-1.46	21.6%	-	-
Seperated/divored/widowed	139 24.3%	1.49**	1.17-1.89	39.5%	1.57**	1.27-1.95

**Employment status**						
Employed/Retired/Student	18.9%	-	-	29.9%	1.05	0.71-1.41
Unemployed	30.6%	1.89**	1.34-2.66	29.8%	-	-

**Socioeconomic status (SES)^2^**						
Low	22.3%	1.38*	1.12-1.72	34.9%	2.32**	1.56-3.44
Medium	17.3%	-	-	28.0%	-	-
High	18.0%	1.05	0.70-1.58	30.8%	0.43**	0.29-0.64

**Self-perceived health status^3^**						
poor	40.6%	4.06**	3.07-5.37	68.8%	9.38**	7.04-12.50
average	30.4%	2.56**	2.02-3.33	48.7%	4.05**	3.21-5.10
good	14.4%	-	-	19.0%	-	-

Logist. Regression, Stress↓ (PSQ < 0,30) vs. Stress↑ (PSQ > 0,30), Fatigue↓ (< 6 months) vs. Fatigue↑ (> 6 months)

*p < 0.05

**p < 0.001

^2 ^Winkler and Stolzenberg (1999)

^3 ^Indicator generated by single-item: „How content are you about your health status?"; poor: not content at all; average: depends; good: content-very content.

Marital status is significantly correlated to both fatigue and stress perception; being divorced or separated increases the probability of both. Employment status contributes only substantially to stress perception not to fatigue.

Respondents with a lower socioeconomic status have significantly higher risk for fatigue than those from middle or high social classes. Poorly rated health is strongly associated with both a high stress perception and fatigue.

## Discussion

We conducted a large survey of fatigue and stress perception in the general population to investigate sociodemographic correlates and the relation between fatigue and perceived stress. Furthermore, this paper presented the prevalence rates of both fatigue and perceived stress according to age and gender. The study showed that perceived stress and fatigue are related constructs. We found a notable association between fatigue and stress. The highest relationship could be detected between fatigue, tension and lack of joy. We conclude that fatigue and stress seem to share overlapping correlates and that aspects of these constructs seem to overlap. Common risk factor patterns could be identified according to socio-economic status and self-perceived health status: the lower the health status the higher the risk for stress and fatigue.

The relation of perceived stress (measured with the PSQ) and psychiatric morbidity was reported elsewhere (3-7) and was not included in the underlying study. Yet it could have helped to distinguish perceived stress from psychiatric morbidity and thus is a limitation of the study.

Studies of fatigue in the past have been based in clinical settings. The high prevalence rates of fatigue support the notion of a continuum of fatigue, as suggested by data from studies in clinical settings [19-25]. In our survey, 25.9% of male and 34.5% of female respondents reported fatigue during the last six months, and 9.7% of the general population reported substantial fatigue lasting six months or longer. These findings confirm the importance of fatigue as a symptom in the general population [2]. The trend of fatigue over a lifetime seems to be linear, while stress perception appears to be bell-shaped.

As in comparable studies, women report higher fatigue than men [21,26]. Other correlates of fatigue have not been reported before; employment status plays no significant role and higher social class lowers the risk of fatigue. A study of functional limitations of fatigued patients in Dutch general practices concluded that limitations from fatigue are rather unspecific and are related to "poor overall health"[2].

## Conclusion

Elevated fatigue and perceived stress overlap most in terms of: low socioeconomic status and poor self-perceived overall health. Fatigue could be a stress-related disorder triggered by long lasting tension. Therefore, this relation deserves more research attention, particularly with regard to *unexplained *fatigue.

## Competing interests

The authors declare that they have no competing interests.

## Authors' contributions

RK participated in the study design, performed statistical analysis and drafted the manuscript. AH participated in the study design and advised for analysis. EB participated in the sequence alignment and acquisition of data. BK conceived of the study, and participated in its design and coordination. All authors read and approved the final manuscript.

The nationwide survey was conducted with the assistance of an institute specialized for demographic research (USUMA, Berlin) which could provide a statement of approval and informed consent for publication from the persons form the general population who participated in the study. A copy of the written consent is available for review by the Editor-in Chief of BMC Research Notes. No experimental research took place in the study.
